# Mechanisms of Endothelial Dysfunction in Antiphospholipid Syndrome: Association With Clinical Manifestations

**DOI:** 10.3389/fphys.2018.01840

**Published:** 2018-12-21

**Authors:** Manuela Velásquez, Mauricio Rojas, Vikki M. Abrahams, Carlos Escudero, Ángela P. Cadavid

**Affiliations:** ^1^Grupo Reproducción, Departamento de Microbiología y Parasitología, Escuela de Medicina, Universidad de Antioquia, Medellín, Colombia; ^2^Grupo de Inmunología Celular e Inmunogenética, Facultad de Medicina, Coordinador Unidad de Citometría de Flujo, Sede de Investigación Universitaria, Universidad de Antioquia, Medellín, Colombia; ^3^Department of Obstetrics, Gynecology and Reproductive Sciences, Yale School of Medicine, New Haven, CT, United States; ^4^Vascular Physiology Laboratory, Group of Investigation in Tumor Angiogenesis (GIANT), Department of Basic Sciences, Universidad del Bío-Bío, Chillán, Chile; ^5^Group of Research and Innovation in Vascular Health (GRIVAS Health), Chillan, Chile; ^6^Red Iberoamericana de Alteraciones Vasculares Asociadas a Transtornos del Embarazo, Chillan, Chile

**Keywords:** antiphospholipid syndrome, endothelial dysfunction, antiphospholipid antibodies, inflammation, thrombosis

## Abstract

The endothelium is a monolayer of cells that covers the inner surface of blood vessels and its integrity is essential for the maintenance of vascular health. Endothelial dysfunction is a key pathological component of antiphospholipid syndrome (APS). Its systemic complications include thrombotic endocarditis, valvular dysfunction, cerebrovascular occlusions, proliferative nephritis, deep vein thrombosis, and pulmonary embolism. In women, APS is also associated with pregnancy complications (obstetric APS). The conventional treatment regimens for APS are ineffective when the clinical symptoms are severe. Therefore, a better understanding of alterations in the endothelium caused by antiphospholipid antibodies (aPL) may lead to more effective therapies in patients with elevated aPL titers and severe clinical symptoms. Currently, while *in vivo* analyses of endothelial dysfunction in patients with APS have been reported, most research has been performed using *in vitro* models with endothelial cells exposed to either patient serum/plasma, monoclonal aPL, or IgGs isolated from patients with APS. These studies have described a reduction in endothelial cell nitric oxide synthesis, the induction of inflammatory and procoagulant phenotypes, an increase in endothelial proliferation, and impairments in vascular remodeling and angiogenesis. Despite these lines of evidence, further research is required to better understand the pathophysiology of endothelial dysfunction in patients with APS. In this review, we have compared the current understanding about the mechanisms of endothelial dysfunction induced by patient-derived aPL under the two main clinical manifestations of APS: thrombosis and gestational complications, either alone or in combination. We also discuss gaps in our current knowledge regarding aPL-induced endothelial dysfunction.

## Introduction

Antiphospholipid syndrome (APS) is an autoimmune disease characterized by a persistence (≥12 weeks) of moderate to high titers of immunoglobulin isotype G (IgG) and IgM antiphospholipid antibodies (aPL) reactive against either cardiolipin (aCL) or β2 glycoprotein I (aβ2GPI); or positive tests for lupus anticoagulant (LA). Clinically, APS is defined as either primary APS, when it occurs in the absence of any other related disease, or as secondary APS, when it is associated with other autoimmune pathologies, such as systemic lupus erythematosus (SLE) (Mackworth-Young et al., 1989). Another variant termed catastrophic APS is characterized by rapid episodes of thrombosis in small vessels of multiple organs causing a systemic dysfunction (Asherson et al., 2003). The clinical manifestations of APS include thrombosis and/or pregnancy complications (Miyakis et al., 2006). Systemic complications of APS include thrombotic endocarditis, valvular dysfunction, cerebrovascular occlusions, proliferative nephritis, deep vein thrombosis, and pulmonary embolism. Pregnancy complications associated with APS, also termed obstetric APS, are characterized by recurrent pregnancy loss, placental insufficiency, preeclampsia, and fetal growth restriction. Some female patients may present with obstetric APS in the absence of systemic thrombosis or purely obstetric APS (Bouvier et al., 2014). In contrast, there are female APS patients with prominent systemic thrombosis in whom it is not usual to find obstetric events. However, APS can present with both pregnancy complications and systemic thrombosis simultaneously. In patients with obstetric APS, pregnancy complications can be triggered by lower titers of aPL than those in patients with thrombotic APS (Meroni et al., 2018). Considering the above, there are several types of patients in which aPL induce different pathological events and thus, distinct mechanisms may be involved.

Patients with APS exhibit endothelial dysfunction. Mechanistically, vascular alterations in patients with APS, including arterial/venous hyperplasia with occlusion and stenosis in several organs (Amigo and García-Torres, 2000; Martínez-Sales et al., 2011), begins with the binding of aPL to the endothelium. This binding involves the participation of membrane receptors that, in some cases, may require the presence of serum β2GPI (Mineo, 2013; Padjas et al., 2016). Indeed, β2GPI is considered to be one of the major pathological autoantigens in APS (Ioannou et al., 2011). However, our understanding of endothelial dysfunction in APS is still limited. While patients with APS are not usually classified by their clinical manifestations, some clinical events indicate a greater association with thrombosis, pregnancy complications, or both. For instance, our group reported that serum from patients with APS exhibiting thrombosis and pregnancy loss simultaneously induced stronger deleterious effects on endothelial function, such as vascular remodeling and angiogenesis, than serum from patients with pregnancy complications alone (Velásquez et al., 2016). Thus, developing a better understanding of the pathophysiology and clinical manifestations of endothelial dysfunction in APS would facilitate the improvement of current treatment regimens, which are ineffective for some groups of patients (Espinosa et al., 2011; Scoble et al., 2011; Mekinian et al., 2015).

In this review, we have compared the current understanding about the mechanisms of endothelial dysfunction induced by patient-derived aPL under the two main clinical manifestations of APS: thrombosis and gestational complications, either alone, or in combination (summarized in Table 1). We also discuss gaps in our current knowledge regarding aPL-induced endothelial dysfunction.

**Table 1:** Summary of the mechanisms of endothelial dysfunction in antiphospholipid syndrome and its association with clinical manifestations.

**Clinical classification**	**aPL**	**Receptor**	**Target protein**	**Pathway**	**Model**	**Patients (gender) and clinical manifestation**	**Effect**	**References**
Obstetric APS	NE-unknown aß2GPI?	NE-unknown	↑ C3/C9	NE-unknown	*In vivo: BALB/c female* *Ex vivo: uterine* endothelial cells	NA	↑ Fetal loss	(Agostinis et al., 2011)
	NE-unknown	NE-unknown	↑ C3a	NE-unknown	*In vivo: BALB/c female*	NS VT and PC	↑Fetal resorption	(Girardi et al., 2004)
Thrombotic APS	aß2GPI	apoER2	↓eNOS	↑PP2A	*In vivo:* C57BL/6 *in vitro:* endothelial cells *an*d monocytes	2 men 2 women VT and 1 patient with PC	↑Monocyte adhesion ↑ Thrombus formation	(Ramesh et al., 2011)
	aß2GPI? alone or aCL dependent on ß2GPI?	apoER2	↓eNOS	↑PP2A Dab2/SHC1 recruitment ↓ p-Akt	*In vivo:* C57BL/6 (apoER2- ^fl/fl^ and apoER2- Δ) *in vitro:* HUVEC and HAEC cells	3 men 1 woman AT with CAPS, VT with CAPS and pulmonary hemorrhage, DVT and renal microthrombotic	↑ Thrombus size ↓ Time to occlusion	(Sacharidou et al., 2018a)
	Monoclonal aCL dependent on ß2GPI (CL15 and IS4) IS4 bind to ß2GPI alone	NE-unknown	↑ MCP-1	NF-κB?	*In vitro*: HUVEC cells	1 man (CL15) 1 woman (IS4) 1 patient with DVT 1 patient with DVT and SLE	↑Monocyte chemotaxis	(Cho et al., 2002)
	aßGPI (human) aß2GPI (rabbit)	Annexin A2	NE-unknown	NE-unknown	*In vitro*: HUVEC and Mono Mac 6 monocytes	NS LA+ and VT	↑Monocyte adhesion	(Zhang and McCrae, 2005)
	NE-unknown	NE-unknown	↑ C5/C3	NE-unknown	*In vivo: C57BL/6 C3^−/−^ C5^−/−^, CD1 male*	NS	↑ Thrombus size ↑Adhesion of leukocytes to endothelium	(Pierangeli et al., 2005)
	NE-unknown	NE-unknown	NE-unknown	NE-unknown (p38 MAPK?)	*In vitro*: HUVEC cells	28 women VT with or without PC	↑Endothelial MP E-selectin+	(Pericleous et al., 2013)
	LA	NE-unknown	NE-unknown	NE-unknown (p38 MAPK?)	*In vitro*: HUVEC cells and endothelial MP of human plasma	30 patients (no gender especified) VT and PC, NS	↑MP (E-selectin +, ICAM-1+ and CD31+)	(Combes et al., 1999)
Thrombotic and obstetric APS	NE-unknown	NE-unknown	↑ C3 convertase	NE-unknown	*In vivo: (BALB/c female, C57BL/6 C3^−/−^ and CD1 male)*	1 patient NS LA+ and aCL+, multiple cerebrovascular accidents, livedo reticularis	↑ Thrombus size ↑Fetal resorption ↓Fetal weight	(Holers et al., 2002)
	NE-unknown	TLRs?	NE- unknown	↑ROS ↑p-ATF-2 ↑p-p38 MAPK	*In vitro*: HUVEC cells and THP-1 monocytes	2 men 10 women DVT, SVT, AT, DVT with or without PC or SLE	↑ROS ↑ VCAM-1	(Simoncini et al., 2005)
	NE-unknown	TLRs?	NE- unknown	↑p-p38 MAPK NF-κB	*In vitro*: HUVEC cells	4 men 4 women DVT and PC	↑TF ↑ IL-8/IL-6 ↑iNOS	(Vega-Ostertag et al., 2005)
	aß2GPI	TLR2 and TLR4	NE-unknown	MyD88 ↑ NF-κB	*In vitro*: HUVEC cells and HMEC-1	NS 1 patient with APS	↑ E-selectin	(Alard et al., 2010; Raschi et al., 2014)
	aß2GPI?	NE- unknown	↑mTORC1 ↑mTORC2	RAPTOR (↑p-S6) RICTOR (↑p-Akt)	*In vitro*: HMEC-1 cells *ex vivo*: renal biopsies	49 men 57 women APS associated to nephropathy with anticoagulant medication	Nephropathies endothelial hyperplasia	(Canaud et al., 2014)
	NE-unknown	NE-unknown	↓MMP-2/9 ↓VEGF	↓ NF-κB	*In vitro*: HEEC cell *In vivo:* CD1 mice	6 women VT, AT with or without PC	↓Angiogenesis	(Di Simone et al., 2010)
	NE-unknown	NE-unknown	VEGF not altered	NE- unknown	*In vitro*: HUVEC and trophoblast cells	10 women VT with PC	↓Angiogenesis ↓Vascular remodeling	(Velásquez et al., 2016)
	NE-unknown	NE-unknown	NE-unknown	NE- unknown	Feto-placental vasculature and *ex vivo*: maternal serum	12 women VT with PC and SLE	Partial villous infarction, thrombosis ↑ ICAM-1 ↑ VCAM-1	(Lakasing et al., 2000)

*NS, not specified; NE, not evaluated; NA, not applicable; aB2GPI, anti- β2 glycoprotein I; apoER2, apolipoprotein E receptor 2; eNOS, endothelial nitric oxide synthase; PP2A, protein phosphatase 2A; VT, vein thrombosis; PC, previous history of pregnancy complications; aCL, anti-cardiolipin, Dab2, disabled 2; SHC1, Scr homology 2 domain containing; p-Akt, protein kinase B; apoER2^fl/fl^, apolipoprotein E receptor 2 floxed; apoER2Δ, apolipoprotein E receptor 2 modified; HUVEC, human umbilical cord vein endothelial cells; HAEC, human aortic endothelial cells; AT, arterial thrombosis; CAPS, catastrophic APS; DVT, deep vein thrombosis; MCP-1, monocyte chemoattractant protein-1; NF-κB, nuclear factor κB; SLE, systemic lupus erythematosus; p38-MAPK, p38 mitogen-activated protein kinases; MP microparticles; LA, lupus anticoagulant; TLRs, Toll-like receptors; ATF, activating transcription factor; ROS, reactive oxygen species; VCAM, vascular cell adhesion protein 1; SVT, superficial venous thrombosis; TF, tissue factor; IL-8/IL-6, interleukins 8 and 6; iNOS, inducible nitric oxide synthase; MyD88, myeloid differentiation primary response 88; HMEC-1, human dermal microvascular endothelial cells; mTORC1, mammalian target of rapamycin complex 1; mTORC2, mammalian target of rapamycin complex 2; RAPTOR, regulatory-associated protein of mTOR; S6, ribosomal protein; RICTOR, rapamycin-insensitive companion of mTOR; MMP-2/9, metalloproteinases 2/9; VEGF, vascular endothelial growth factor; HEEC, endometrial endothelial cells; ICAM, intercellular adhesion molecule 1. Question marks indicate current gaps in our knowledge*.

## Impaired Synthesis of Antithrombotic Factors in APS

**Figure 1:**
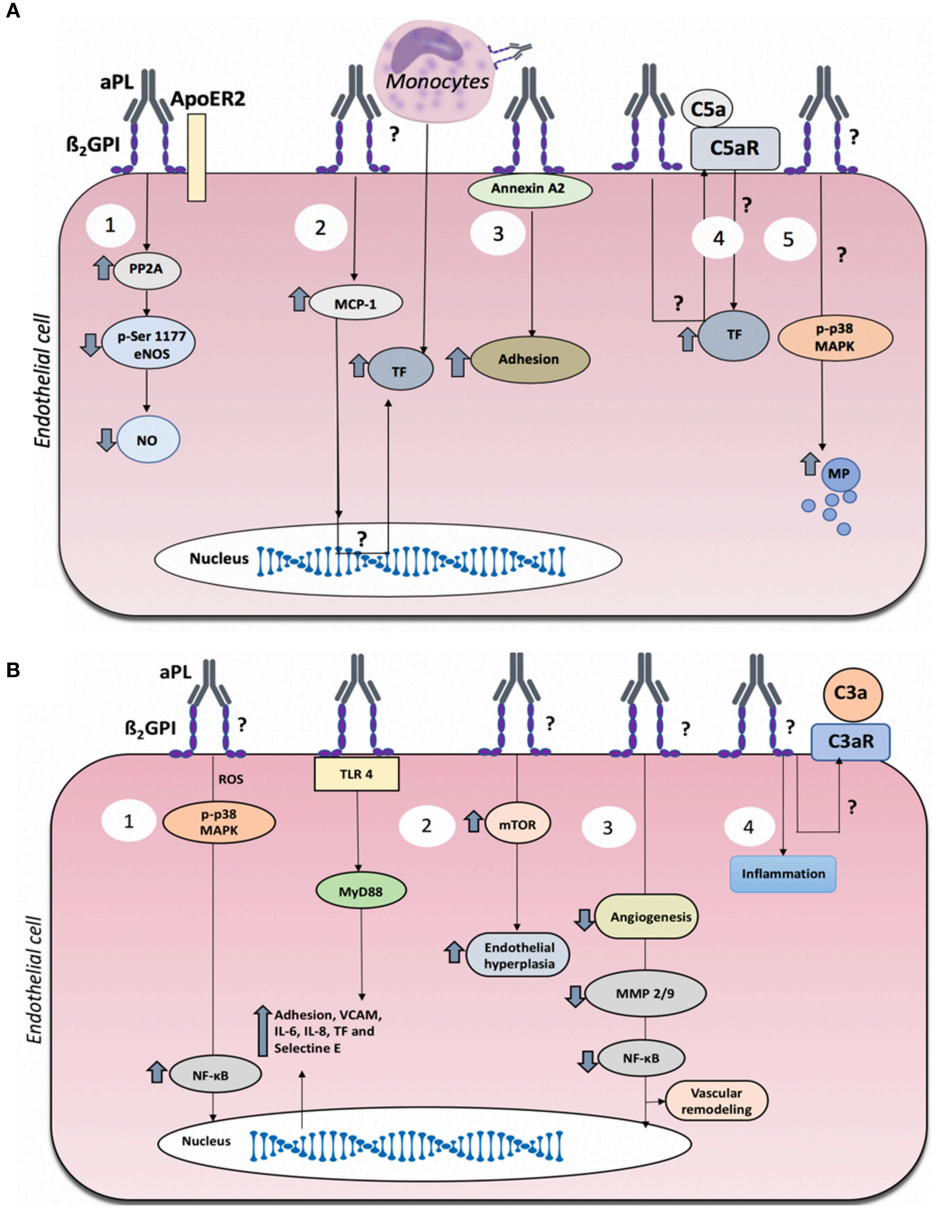
Mechanisms of endothelial dysfunction in antiphospholipid syndrome (APS). **(A)** Mechanisms of endothelial dysfunction associated with thrombosis in APS. Thrombotic events in APS may be associated with several events triggered by aPL: (1) Reduced nitric oxide generation via ApoER2. (2) Elevated endothelial cell production of MCP-1, which favors the adhesion of monocytes to the endothelium, resulting in increased TF. (3) Binding of anti-β2GPI antibodies to annexin A2/β2GPI complexes on the plasma membrane induces elevated expression of adhesion molecules. (4) Complement C5a generation which in turn induces TF expression. (5) Increased production of endothelial microparticles (MP). **(B)** Mechanisms of endothelial dysfunction associated with obstetric APS with or without thrombosis. Obstetric APS may be associated with several events triggered by aPL: (1) Induction of inflammation via the TLR, MyD88, MAPK, and NF-κB pathways. (2) mTOR-mediated endothelial proliferation. (3) reduced maternal vascular remodeling. (4) inflammation and placental damage associated with complement activation.

Impaired synthesis of the vasodilatory factor, nitric oxide (NO), has been described in patients with APS. Patients with APS displaying thrombosis exhibited low plasma levels of nitrites and nitrates, which are the stable metabolites of NO breakdown. Clinically, these low levels were associated with vascular occlusions, suggesting an enhanced risk of thrombotic, and inflammatory events (Ames et al., 2010). Additionally, aPL can act as endogenous antagonists of endothelial nitric oxide synthase (eNOS) through β2GPI, and this interaction may impair NO synthesis. In particular, attenuation of eNOS activation by aPL was mediated by reduced phosphorylation of eNOS serine 1179. This inhibition of eNOS phosphorylation was shown to be dependent upon protein phosphatase 2A (PP2A), β2GPI, and apolipoprotein E receptor 2 (ApoER2) (Figure 1A) (Ramesh et al., 2011; Sacharidou et al., 2018a). Furthermore, aPL inhibition of eNOS activity contributes to thrombus formation, increased leukocyte adhesion, and alterations in vascular tone. A pro-thrombotic phenotype of platelets and monocytes also appears to be triggered by aPL through ApoER2 (Sacharidou et al., 2018b). It remains unclear whether such apoER2-PP2A-eNOS activation/deactivation occurs in all clinical manifestations of APS or only in the more severe conditions associated with thrombosis. Additionally, it has not yet been clarified whether this pathway is activated in patients with obstetric APS. Therefore, there is a need for further studies that include patients with obstetric APS alone as a control.

The regulation of vascular tone mediated by other molecules in APS patients is an area worthy of research since we were unable to find any evidence in the literature of the assessment of endothelial control of vascular tone in APS patients. Therefore, this, and the participation of other vasoactive substances, including prostacyclin or other arachidonic acid metabolites, endothelin-1, or purinergic metabolites might be the focus of future research. aPL can induce an imbalance in the production of thromboxane and prostacyclin that favor platelet activation, but the mechanism by which aPL alters the endothelial production of prostacyclin is unclear, since the reported results are controversial (Lellouche et al., 1991; Lindsey et al., 1992).

Another potential contributor to thrombosis formation in APS involves heparan sulfate, which is required for the adequate anticoagulant activity of anti-thrombin (Moon et al., 2005). β2GPI binds to heparan sulfate via an anionic-cationic interaction, thus offering epitopes for circulating aPL (Meroni et al., 1998). The intracellular consequences of the interaction between heparan sulfate-bound β2GPI and aPL have yet to be determined. However, in other autoimmune conditions, such as SLE and in patients with recurrent thrombosis, the functional activity of anti-thrombin is lost in the presence of antibodies against heparan sulfate (Cosgriff and Martin, 1981; Fillit et al., 1993). It is unclear how those changes are releted with endothelial dysfunction.

## Increased Interactions Between the Endothelium and Monocytes

In APS, increased interactions between monocytes and endothelial cells are associated with thrombosis (Clemens et al., 2009). aPL can directly activate monocytes, which in turn interact with the endothelium, resulting in pro-thrombotic events, such as the production of tissue factor (TF) (Kinev and Roubey, 2008; Shantsila and Lip, 2009). Conversely, the exposure of endothelial cells to aPL upregulates endothelial expression of monocyte chemoattractant protein 1 (MCP-1), which in turn promotes TF synthesis by monocytes (Figure 1A) (Cho et al., 2002), leading to thrombotic complications in patients with primary APS (Cuadrado et al., 1997).

In the presence of β2GPI, aCL directly induce activation of the mitogen-activated protein kinase (MAPK) pathway in monocytes. These aPL stimulated phosphorylation of p38 MAPK, nuclear factor κB (NF-κB) translocation to the nucleus, and the upregulation of TF. The aPL-induction of monocyte TF could be prevented by the p38 MAPK inhibitor SB203580 (Yasuda et al., 2005). Interestingly, IgG from patients with APS induced monocyte TF expression and adherence to endothelial cells *in vitro*, and in an *in vivo* mouse model induced thrombosis and leukocyte adherence; all via p38 MAPK activation (Vega-Ostertag et al., 2007). aβ2GPI exhibit high-affinity binding to other molecules, such as annexin A2 that is expressed on the surface of endothelial cells. Annexin A2 serves as an anchor for the binding of phospholipids and aPL, especially aβ2GPI, as described in a cohort of LA-positive patients with a history of venous thrombosis. Although the participating intracellular pathways remain unknown, endothelial activation evidenced by increased monocyte adhesion was observed after aβ2GPI/annexin A2 binding (Figure 1A) (Zhang and McCrae, 2005).

In summary, the adhesion of monocytes to the endothelium is pivotal in mediating aPL-induced TF production and formation of thrombotic clots. Additionally, the adhesion of monocytes to the endothelium would be expected to potentiate inflammation. However, there has been no clear association between this event and the clinical characteristics of patients so far, mainly because the reports have not included a control group of patients with obstetric APS.

## Prothrombotic Endothelial Microparticles Released in APS

Endothelial dysfunction is associated with the synthesis of endothelial extracellular particles, such as microvesicles (Morel et al., 2006; Pericleous et al., 2009). *In vitro* studies demonstrated a significant increase in the release of microparticles from endothelial cells cultured in the presence of IgG from patients with APS and clinical manifestations of thrombosis with or without pregnancy complications, when compared to control IgG. In contrast, aPL from patients with pregnancy complications, but without thrombosis, did not significantly alter endothelial microparticle release (Pericleous et al., 2013). Another study showed an increase in the production of microparticles expressing pro-adhesive and pro-coagulant proteins in endothelial cells stimulated with plasma from patients positive for LA, some of whom also had APS (Combes et al., 1999).

In APS, the molecular pathways involved in the aPL-induction of endothelial microparticle release have not been determined. However, generation of these particles seems to be dependent on p38 MAPK activation (Figure 1A) (Curtis et al., 2009), and thus based on our previous discussion could favor thrombotic episodes. Since the study of circulating microparticles allows both the detection of biomarkers for disease risk factors and the identification of intercellular communication mechanisms that contribute to pathological states, future studies focusing on the role of these microparticles in patients with APS are of high priority.

## Complement-Dependent Endothelial Activation in APS

aPL trigger activation of the complement cascade generating the active components, C3a, C3b, and C5a. C5a is a potent anaphylatoxin that can induce thrombosis through chemotactic actions (Erkan and Salmon, 2016). Endothelial cells express complement receptors, such as C5aR that interacts with C5a. The binding of this receptor to its ligand induces TF production (Wojta et al., 2002). Using C3, C5, and C5aR deficient mice, as well as blocking anti-C5 monoclonal antibodies, aPL-induced endothelial activation and thrombosis was shown to be complement dependent (Figure 1A) (Pierangeli et al., 2005; Vega-Ostertag et al., 2007). Similarly, when the complement component C3 convertase was inhibited in an experimental model, the induction of thrombosis, fetal resorption, and low fetal weight by aPL was reduced. This indicates the relevance of complement in the pathophysiology of APS featuring both thrombosis and pregnancy complications (Figure 1B) (Holers et al., 2002). However, despite the strong experimental association between aPL-mediated complement activation, endothelial dysfunction, and thrombus formation, there remains a lack of strong clinical data to support these mechanistic relationships. In contrast, a clinical study has shown that complement activation early in pregnancy is predictive of adverse pregnancy outcomes in women with aPL (Kim et al., 2018).

## Production of Proinflammatory Factors in Patients With APS

Endothelial cells treated with IgG-aPL from men and women with different clinical manifestations of APS showed increased reactive oxygen species (ROS) generation, which resulted in increased endothelial expression of vascular cell adhesion molecule-1 (VCAM-1) via p38 MAPK activation (Simoncini et al., 2005). In another study that examined aPL from men and women with clinically active APS, IgG-aPL treatment of endothelial cells activated the cells to express increased TF, interleukin-6 (IL-6), and IL-8. These prothrombotic and proinflammatory responses were mediated by activation of the p38 MAPK and NF-κB pathways (Figure 1B) (Vega-Ostertag et al., 2005). Unfortunately, in these studies, the patients were not classified by gender or clinical manifestation, which hinders the precise understanding of the mechanisms described in association with the pathophysiology of APS.

One mechanism by which aPL may mediate endothelial cell inflammation is through activation of the innate immune receptors, Toll-like receptor 2, and 4 (TLR2 and TLR4). Dimeric β2GPI can bind TLR2 and TLR4, which favor aPL binding (Alard et al., 2010; Raschi et al., 2014). aPL with β2GPI reactivity from patients with thrombosis and pregnancy complications induced endothelial cell activation through activation of MyD88, a key component of the TLR2, and TLR4 signaling pathways (Figure 1B). Notably, β2GPI shares molecular mimicry with certain pathogenic microorganisms that can activate TLR4 (Raschi et al., 2003). However, further studies are needed whereby patients are grouped according to their clinical manifestations and gender to obtain a more detailed understanding of the pathological effects of aPL on the endothelium.

## Endothelial Dysfunction in Women With Obstetric APS

In cases of obstetric APS there is endothelium involvement, despite the often lack of thrombotic events (Viall and Chamley, 2015). aPL specific for β2GPI are the most pathologic obstetrically by targeting the uterine endothelium, as well as the placental trophoblast and endometrial/decidual stroma, where high basal levels of β2GPI are normally expressed (Agostinis et al., 2011; Meroni et al., 2018). Unlike systemic APS, which is a thrombotic disease, obstetric APS is associated with inflammation at the maternal-fetal interface, and poor placentation associated with reduced trophoblast invasion and limited uterine spiral artery remodeling (Viall and Chamley, 2015). Thus, patients with obstetric APS may present a fundamentally different disease in contrast to patients with thrombotic APS; and the same aPL might induce both clinical conditions through these distinct mechanisms (Meroni et al., 2018).

In some women with obstetric APS who do not experience thrombosis, placental damage has been associated with inflammatory events (Asherson et al., 2006). Placental injury in obstetric APS has also been associated with complement activation (Figure 1B) (Alijotas-Reig, 2010). Moreover, in a murine model of obstetric APS it was demonstrated that heparin prevented gestational complications through the inhibition of complement activation (Girardi et al., 2004).

While there has been an abundance of studies showing that aPL deleteriously affect placental function (Stone et al., 2006; Tong et al., 2015), how aPL affect uterine endothelial function is a significantly understudied area. In particular, studies using aPL from patients with pregnancy morbidities in the absence of thrombosis are warranted.

## Vascular Remodeling and Angiogenesis in APS: Implications for Placentation

Endothelial cells are key components of the angiogenesis process. For instance, a study using uterine endothelial cells showed that aPL from women with APS reduced *in vitro* angiogenesis when compared to serum from normal subjects (D'Ippolito et al., 2012). The authors did not detail the clinical characteristics of the patients, making it unclear whether the alterations in angiogenesis caused by aPL may be associated with thrombosis, pregnancy complications or both. Angiogenesis requires the degradation of the vascular basement membrane and remodeling of the extracellular matrix through the production of matrix metalloproteinases (MMP) (Han et al., 2001). *In vivo* angiogenesis assays were used by Di Simone et al. (2010) to document impaired angiogenesis of aPL following the subcutaneous implantation of angioreactors in the dorsa of CD1 mice. The expression of the MMP-2/-9 was decreased in presence of aPL and this was associated with a reduced activation of NF-κB (Figure 1B) (Di Simone et al., 2010).

On the other hand, impaired angiogenesis in women with APS may have profound implications in pregnancy, since placentation requires new vessel formation. Currently, it is unclear whether APS-associated defects in angiogenesis during placental development is more severe in patients with thrombosis who experience pregnancy complications.

Vascular remodeling of the uterine spiral arteries by the invading extravillous trophoblast is another important process of normal placentation. In obstetric APS, endothelial-trophoblast interactions are impaired, leading to reduced uterine spiral artery remodeling (Viall and Chamley, 2015; Silva and Serakides, 2016). In our previous studies we used an *in vitro* model of uterine spiral artery remodeling in which we could measure vessel-like tube structures after the co-culture of first trimester extravillous trophoblast cells with either human endometrial endothelial cells or human umbilical cord vein endothelial cells (Alvarez et al., 2015; Velásquez et al., 2016). In these studies we found that serum from women with obstetric APS either with or without thrombosis decreased the trophoblast-endothelial interactions and thus, the formation of stable vessel-like tube structures. In the feto-placental vasculature of women with APS and SLE, findings of partial villous infarction, intravascular thrombosis, and fibrin deposits associated with increased levels of intercellular adhesion molecule-1 and vascular cell adhesion molecule-1 have also been reported (Lakasing et al., 2000).

Future studies, however, are still needed to better understand how aPL impair placental angiogenesis and to elucidate the clinical consequences. Additionally, *in vitro* analyses using serum or IgG-aPL, must consider the clinical parameters and gender of the included patients.

## Proliferation Markers Associated With Endothelial Hyperplasia

In renal biopsies from patients with APS, the endothelium exhibits activation of mTOR, a kinase involved in the regulation of cell proliferation. To detect the activation of mTOR (mTORC1 or mTORC2), the phosphorylation of ribosomal protein S6 and serine 473 of Akt in endothelial cells of the renal vasculature was evaluated. Among the several interesting findings from this study, we highlight the following. First, aPL isolated from patients with APS induced mTOR activation in endothelial cells *in vitro*. Second, treatment with rapamycin inhibited mTOR activation via RAPTOR and RICTOR. Third, in patients with catastrophic APS, the same mTOR activation was observed in the renal endothelium and was accompanied by severe vascular constriction. Finally, in patients with APS and renal transplantation who were treated with rapamycin, mTOR activation was not detected and the patients showed reduced renal lesions and an absence of endothelial hyperplasia between 3 and 12 months after transplantation. The authors concluded that the mTOR signaling pathway is involved in the development of the endothelial dysfunction that result in the clinical manifestations of APS (Figure 1B) (Canaud et al., 2014). However, these authors did not specify the clinical characteristics of their patients with APS and, therefore, it is unclear whether this pathological mechanism of aPL is associated with thrombosis, pregnancy complications or both. These results reinforce necessity of better clinical characterization of subjets to better pathophysiology understanding.

## Conclusion

Patients with APS are classified into different groups depending on the severity of their clinical manifestations and on their aPL titers. Endothelial dysfunction is a key component of APS. However, our understanding of the precise mechanisms by which aPL induce endothelial dysfunction remains limited, in part, because patients are not always classified according to their clinical manifestations. Nonetheless, some mechanistic events do indicate a greater association with thrombosis, pregnancy complications, or both. In Table 1, we summarize the gaps in knowledge to highlight research topics that warrants further attention. Future studies involving patients with APS should consider clinical characteristics and gender to better understand the pathophysiology of endothelial dysfunction in this disease.

## Author Contributions

MV wrote the draft of the manuscript. MR, VA, CE, and ÁC critically revised the manuscript. ÁC generated the original idea and proposed topics for revision.

### Conflict of Interest Statement

The authors declare that the research was conducted in the absence of any commercial or financial relationships that could be construed as a potential conflict of interest.

## Abbreviations

aPL: antiphospholipid antibodies
APS: antiphospholipid syndrome
ApoER2: apolipoprotein E receptor 2
obstetric APS: APS in pregnancy complications
aCL: antibodies anti- cardiolipin
C3a, C3b, and C5a: complement cascade active components
eNOS: endothelial nitric oxide synthase
IgG: immunoglobulin isotype G
IgM: immunoglobulin isotype M
IL-6: interleukin-6
IL-8: interleukin-8
LA: lupus anticoagulant
MMP-2: matrix metalloproteinases 2
MMP-9: matrix metalloproteinases 9
MAPK: mitogen-activated protein kinase
MCP-1: monocyte chemoattractant protein 1
mTOR: mammalian target of rapamycin complex
NF-κB: nuclear factor κB
NO: nitric oxide
PP2A: protein phosphatase 2A
RAPTOR: regulatory-associated protein of mTOR
S6, RICTOR: ribosomal protein
SLE: systemic lupus erythematosus
TF: tissue factor
TLR2: toll-like receptor 2
TLR4: toll-like receptor 4
VCAM-1: vascular cell adhesion molecule-1
aβ2GPI: antibodies β2 glycoprotein I.
